# Aberrant Oligodendrogenesis in Down Syndrome: Shift in Gliogenesis?

**DOI:** 10.3390/cells8121591

**Published:** 2019-12-07

**Authors:** Laura Reiche, Patrick Küry, Peter Göttle

**Affiliations:** Department of Neurology, Medical Faculty, Heinrich-Heine-University, 40225 Düsseldorf, Germany; Laura.Reiche@hhu.de (L.R.); Patrick.kuery@uni-duesseldorf.de (P.K.)

**Keywords:** down syndrome, white matter, glial fate

## Abstract

Down syndrome (DS), or trisomy 21, is the most prevalent chromosomal anomaly accounting for cognitive impairment and intellectual disability (ID). Neuropathological changes of DS brains are characterized by a reduction in the number of neurons and oligodendrocytes, accompanied by hypomyelination and astrogliosis. Recent studies mainly focused on neuronal development in DS, but underestimated the role of glial cells as pathogenic players. Aberrant or impaired differentiation within the oligodendroglial lineage and altered white matter functionality are thought to contribute to central nervous system (CNS) malformations. Given that white matter, comprised of oligodendrocytes and their myelin sheaths, is vital for higher brain function, gathering knowledge about pathways and modulators challenging oligodendrogenesis and cell lineages within DS is essential. This review article discusses to what degree DS-related effects on oligodendroglial cells have been described and presents collected evidence regarding induced cell-fate switches, thereby resulting in an enhanced generation of astrocytes. Moreover, alterations in white matter formation observed in mouse and human post-mortem brains are described. Finally, the rationale for a better understanding of pathways and modulators responsible for the glial cell imbalance as a possible source for future therapeutic interventions is given based on current experience on pro-oligodendroglial treatment approaches developed for demyelinating diseases, such as multiple sclerosis.

## 1. Introduction

The majority of central nervous system (CNS) diseases are characterized by neuronal damage and white matter malfunctions, which can lead to detrimental motor and sensory effects. Trisomy 21, as an aneuploidy disorder, is characterized by an additional copy of human chromosome 21 (Hsa21) and causes Down syndrome (DS). DS is the most abundant human trisomy, affecting around 1 in 1100 neonates annually [1], making it the most common genetic cause for intellectual disability (ID) [2]. DS patients suffer from several cognitive impairments, accompanied by a low intelligence quotient (IQ) ranging from 30 to 70 [2], which can be attributed to brain abnormalities. In accordance with the neurocentric paradigm, brain research in DS has followed the concept that neuronal dysfunctions primarily lead to neurological diseases [3]. Therefore, much of the DS research aimed at identifying the underlying genetic interventions of altered neurogenesis. This information is essential for unraveling pharmacological approaches to ameliorate cognitive function (summarized in recent reviews [1,4,5,6,7,8]). Nevertheless, over the last few years consideration has been given to the re-evaluation of the role of astroglial and oligodendroglial lineage cells in CNS pathologies characterized by neurodegeneration [3,9]. Interestingly, several studies in DS indicated a neuro- to gliogenic shift, mainly focusing on the observed bias toward astrocytes [3,4,6,10]. Even though oligodendroglial cells—as a source of CNS myelin sheaths—are essential for higher brain functions by assuring long-term axonal integrity, metabolic and trophic support, and accelerated electrical signal propagation, this crucial cell population has not attracted much attention in DS. The notion that aberrant oligodendrogenesis may contribute to cognitive impairments and ID in DS is supported by a recent developmental transcriptome analysis of post-mortem human DS brains [11]. Of note, the analysis of this study revealed a dysregulated gene cluster associated with oligodendroglial cell differentiation and myelination, showing that hypomyelination in DS is caused by a cell-autonomous phenomenon in oligodendrocyte development. To further highlight the importance of the oligodendroglial lineage in DS development, this review article summarizes the current knowledge regarding altered oligodendrogenesis and white matter malformations in human and rodent DS research. We show that signaling pathways assumed to lead to defective neurogenesis and to a neuro-to astrogenic shift also affect oligodendrogenesis. Such knowledge may help to devise new treatments that aim to improve brain development and ID by stabilization of the oligodendroglial lineage.

## 2. Down Syndrome: A Brief Neurological Profile

Associated with more than 80 clinical features affecting many organs, both the occurrence (penetrance) and severity (expressivity) of phenotypes vary across the DS population [4]. Nonetheless, certain characteristics, such as facial dysmorphology, reduced brain volume accompanied by ID, and an early-onset Alzheimer’s disease (AD)-like pathology are common in all DS individuals. This neurological profile is distinctly marked by hypocellularity in the cerebral hemispheres, frontal lobe, temporal cortex, hippocampus, and cerebellum, most likely explained by a complex spatiotemporal perturbation in neurogenesis, resulting in a reduced neuronal cell population and a subsequently altered neuronal connectivity [1,4,6].

Moreover, aberrant astrogliogenesis and changes in several astrocytic marker expression patterns have been demonstrated in DS (reviewed in [3]). Notably, an over-population of astroglial cells in the frontal lobe of DS fetuses [12], as well as in the frontal cortex, calcarine cortex, and mainly hippocampus of infant and adult DS brains [13], has been observed. At an advanced age, astrogliosis in the amygdala [14], related to the occurrence of senile plaques and neurofibrillary tangles [13] and in areas of basal ganglia calcification [15], was shown to be implicated in DS.

Furthermore, DS brains of old adults are marked by reduced numbers of oligodendrocytes when compared to age-matched individuals [16]. More devastating is the observed hypomyelination in DS, pointing to an impaired myelination process which proceeds until adulthood, as demonstrated by myelin protein expression [11], histological [17,18], or magnetic resonance imaging (MRI) [19] examinations. Assessed by diffusion tensor imaging (DTI) fractional anisotropy (FA) analysis, white matter in DS patients showed lower fiber density, smaller axonal diameters, and a reduced myelination degree compared to healthy controls [20]. Decreased FA and early white matter damage were particularly observed in the region of the anterior thalamic radiation, the inferior fronto-occipital fasciculum, the inferior longitudinal fasciculum and the corticospinal tract, bilaterally, the corpus callosum (CC), and the anterior limb of the internal capsule [21,22,23]. Of note, diminished white matter integrity in DS was associated with poorer performance at neuropsychological assessments [20,23]. In this context, recent evidence in animal models suggests that ongoing myelin remodeling is important for behavior, cognition, and learning throughout adulthood [24,25]. Notably, the onset of cognitive deficits in DS is thought to occur in late infancy, becoming more obvious in adolescence [11,26,27,28,29,30,31,32,33]. This time course indeed correlates with the peak of myelination during the first years of life, continuing into young adulthood [34]. Moreover, immunohistochemical analysis for myelin basic protein (MBP) revealed a decreased density of myelinated axons and a generally delayed myelin formation in DS compared to age-matched controls [18], indicating that the oligodendroglial lineage was directly affected upon gene-dosage effects of Hsa21. Accordingly, a recent multi-region transcriptome analysis of DS and healthy brains spanning from fetal development to adulthood revealed that genes associated with oligodendroglial cell differentiation and myelination are dysregulated in trisomy 21 during late fetal development and the first years of postnatal life [11]. Weighted-gene co-expression network analysis (WGCNA) within this study identified several modules of co-expressed genes, including the module number 43 (M43) which is related to oligodendrocyte development and myelination including, for example, 2′,3′-cyclic nucleotide-3′-phosphodiesterase (CNPase), proteolipid protein (PLP), Sox10, and G protein coupled receptor 17 (GPR17). This module exhibited a distinct downregulation throughout the DS neocortex and hippocampus during development [11]. Of note, GPR17, a modulator of oligodendroglial cell maturation [35], is linked to a significantly reduced expression of sorting nexin family member 27 (SNX27) in DS [36], which was demonstrated to impair oligodendroglial precursor cell (OPC) maturation, resulting in myelination deficits in Ts65Dn mice, a mouse model for DS [37]. However, there is much evidence on aberrant oligodendrogenesis correlating with or contributing to DS-related cognitive impairments, but the underlying mechanisms have so far not been investigated in detail.

## 3. Gliogenesis and Cell Types in Healthy CNS

The mammalian central nervous system (CNS) consists of neurons and glial cells, the latter of which make up at least 50% of human brain cells. Glial cell function is essential for the evolutionary increase in complexity of neurological function in mammals [38] and can be divided in macroglial cells deriving from the neuroepithelium and microglia with a hematopoietic (mesodermal) origin [38]. Despite their crucial importance for various physiological processes [39,40], these cells are not further addressed in this review article. Macroglial cells are generally categorized into astrocytes and oligodendrocytes. Due to upcoming knowledge about the functions of proteoglycan nerve-glial antigen 2 (NG2) expressing glial cells, NG2 glia are considered a further category of macroglia [41].

Approximately 40% of the human brain is considered to be white matter. It consists of (i) axons, the functional unit of neurons providing the basis for signal transduction and information, (ii) astrocytes, which are essential for structural and metabolic support to neurons, and (iii) myelin. In the CNS, myelin is imperative for the stabilization, protection, and electrical insulation of axons, enabling accelerated electrical signal propagation [34,35,36]. Myelin sheaths are generated by oligodendrocytes. These specialized glial cells either derive from oligodendroglial precursor cells (OPCs) or niche-located neural stem cells (NSCs) [37]. The structural integrity of myelin is of crucial importance for CNS function and restoration [38]. Unfortunately, pathological degeneration and inflammation [35] or genetic intervention [39] can result in myelin loss, which may lead to impaired neuronal signaling, functional deficits, and a shortened lifetime [40]. Hence, white matter deficits and myelin dysfunctions are considered to be a main contributing factor for neurodegenerative diseases and malfunctions of the CNS [41].

**Figure 1:**
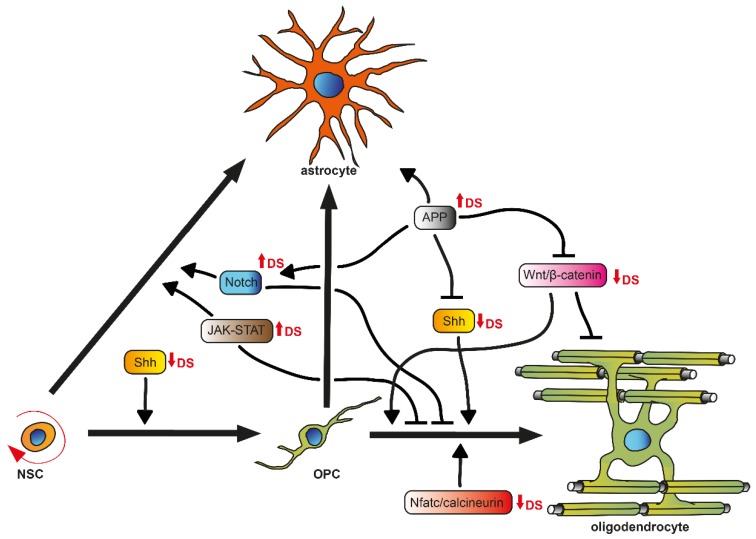
Representation of key signaling pathways involved in oligodendrogenesis. Neural stem cells (NSCs) exhibit astrogenic and oligodendrogenic potential. For oligodendroglial precursor cells (OPCs) derived from NSCs to successfully differentiate into myelinating oligodendrocytes, OPCs follow a highly regulated differentiation process that is affected by a fine-tuned network of signaling pathways. Within Down syndrome (DS) (red arrows), signaling pathways reveal aberrant dynamics.

The major cell types of the CNS are produced by several spatiotemporal, partially overlapping generation and division waves of progenitor cells, which are guided by extrinsic and intrinsic cues [38,42], resulting in a well-defined brain anatomy and cytoarchitecture. In the oligodendrogenic context, it is important to briefly introduce OPCs/NG2 glia and their differentiation potency (Figure 1). These cells derive from radial glia, the primary progenitor cells at embryonic stages, and are produced in three waves following a ventral-dorsal temporal progression in the developing forebrain [38]. They populate the brain and spinal cord to generate oligodendrocytes that myelinate the entire CNS during postnatal life [34]. A small fraction of OPCs is maintained as an immature, slowly proliferative, or quiescent cell population in the adult CNS [43]. Noteworthy, accumulating evidence indicates that beyond generating oligodendrocytes, OPCs exhibit the potential to also give rise to astroglial cells in vitro [44] and in vivo [45,46,47]. As mentioned above, OPCs can additionally be generated postnatally and in the adult brain from transient amplifying cells (TAPs or C cells) derived from NSCs located in the subventricular zone (SVZ) [48], mainly from the dorsal part (facing the corpus callosum) [49]. For OPC differentiation and subsequent myelination to occur, various signals are necessary in order to stabilize oligodendroglial fate and to regulate extensive changes in cell shape and membrane architecture. Pro-oligodendroglial extracellular signals comprise several pathways, such as those elicited by sonic hedgehog (SHH), Wnt/β-catenin, bone morphogenic protein (BMP), cytokines (LIF, Cxcl1), neurotransmitters (glutamate, ATP, adenosine), hormones (thyroid hormone T3, insulin), extracellular matrix molecules (fibronectin, laminin), metabolic signals (hypoxia), or in response to physical cues (spatial constrain, rigid substrate), and axonal receptors (Lingo-1, PSA-Ncam) [9,50,51]. Additionally, intrinsic regulators, such as the transcription factors basic helix-loop-helix oligodendrocyte lineage transcription factor 2 (Olig2) and sex determining region Y-Box 10 (Sox10), have also been implicated in OPC differentiation [52] in that, for example, exposure to SHH, expressed by the ventral telencephalon, instructs early progenitor cells to become OPCs, possibly via upregulation of Olig2 [53]. This induction is antagonized by the dorsally expressed Wnt/β-catenin and BMP pathways [54]. BMP4, on the other hand, has been shown to promote the expression of a family of inhibitor of DNA-binding (Id) proteins Id2 and Id4, which form complexes with Olig2. This interaction prevents Olig2 from binding to DNA, blocking its ability to act as a transcription factor and therefore inhibiting the differentiation along the oligodendroglial lineage but promoting astrogliogenesis [55]. Furthermore, post-translation processes, such as the regulation of the JAK/STAT3 activity by modulating STAT3′s acetylation state, mediated by the histone deacetylase Hdac3, which has been shown to control Olig2 expression, are also needed to suppress astrogliogenesis [56].

Hence, based on this fine-tuned regulation of cell fate and differentiation mediators, it is likely that neurogenesis and gliogenesis are misguided in their responses to gene-dosage abnormalities caused by aneuploidy disorders. This holds true for DS brain development, where not only altered progenitor cell proliferation and apoptosis but also potential signaling pathways responsible for a neuro- to astrogenic shift are assumed to be responsible for observed neuronal hypocellularity and concurrent over-population of astroglial cells. However, so far, an oligodendrogenic to astrogenic shift has not been taken into account, although the number of oligodendrocytes and myelination rate are decreased in DS [11,16,18]. Furthermore, the differentiation of OPCs to mature oligodendrocytes was shown to be negatively affected in the DS mouse model Ts65Dn [11].

## 4. Defective OPC Differentiation in DS—Possible Interfering Regulators

Overall brain volume reduction and hypocellularity are already present in fetuses and children with DS [57,58,59,60]. This fact indicates that defective neuro- and gliogenesis during early phases of brain development may be a major causal factor of DS-associated brain abnormalities, which might be a consequence of early apoptosis and impaired proliferation in DS [6,61]. Also, the comparison of hippocampal regions of DS fetuses between 17 and 21 weeks of gestation to age-matched controls showed a higher percentage of cells with astrocytic phenotypes, but a smaller percentage of cells with neuronal phenotypes [62]. A few studies demonstrated elevated numbers of Olig2 expressing cells (thus declared as OPCs) in DS fetal brains at 14 and 18 weeks of gestation [63], even up to 34 weeks [10], suggesting a cell-fate shift from neurogenesis to gliogenesis at early developmental stages. Of note, the number of Olig2 expressing cells in the DS mouse model Ts65Dn is increased at embryonic day 13.5 and 14 [64], but decreases thereafter [11] when compared to age-matched controls. Intriguingly, the percentage of mature oligodendrocytes was drastically diminished from postnatal days 15–60 [11], whereas a massive increase of astrocyte numbers and reactivity was shown at the age of 48 weeks in the same DS mouse model [65]. Considering the capacity of OPCs to generate astroglial cells instead of oligodendrocytes, a shift within the glial cell commitment in DS accompanied with a generally defective differentiation capacity of oligodendroglial progenitors might be suggested.

Several authors have already discussed the involvement of pathways essential for cell fate and differentiation within neurogenesis in DS, thereby giving strong evidence that therapeutic approaches targeting these pathways could improve aberrant brain cytoarchitecture, in particular the neuro- to astrogliogenic shift [4,5,6,7,61]. Hereinafter, we focus on pathways relevant for oligodendrogenesis (Figure 1) instead and highlight to what extent they might constitute new therapeutic avenues.

### 4.1. JAK-STAT Signaling

One of the most important signaling pathways for the gliogenic cascade in NSCs is the Janus kinase-signal transducer and activator of transcription (JAK-STAT) pathway, mediated by ligands such as interleukins (ILs), interferons (INFs), the glycoprotein (gp) 130 family, and the γ-chain (gC) family [6,66]. Common downstream targets, such as GFAP and S100β which specify glial cell fate, are transcriptionally activated by STATs [6,67]. STAT3 in particular plays an essential role in regulating astrogliogenesis during brain development [66]. In vivo studies showed that overexpression of STAT3 in the neocortex of DS mice (Ts1Cje) enhanced astrogliogenesis [68], whereas its knockout inhibited the astroglial fate in mouse NSCs [69]. Additionally, it was reported that IL-6 in DS children and IFN-γ in embryonic trisomy 16 mouse brains (Ts16, a model used for human trisomy 21 (DS)), were increased respectively [70,71], both of which are capable of activating the STAT3 pathway [72]. Indeed, neonate DS mice (Ts65Dn) exhibited hyperactivation of STAT3 in the hippocampus [73]. More importantly, four IFN receptors, IFN-α receptor 1 and 2 (IFNAR1, IFNAR2), IFN-γ R2 (IFNGR2), and interleukin 10 receptor β (IL10RB), are located on Hsa21 and overexpressed in DS with a mean ratio of ~1.5 proportional to the gene-dosage effect of trisomy 21 [74,75,76]. This disposition leads to a generally increased INF sensitivity in DS [74].

Overstimulation of the JAK-STAT signaling pathway can also be linked to dual-specificity tyrosine-(Y)-phosphorylation-regulated kinase 1A (DYRK1A), which is also overexpressed in DS due to the location on Hsa21 [77]. Overexpression of this protein was shown to result in elevated STAT3 activity, which promoted the astrocytic differentiation of neocortical progenitors in Ts1Cje mice [68].

Interestingly, the STAT3 pathway was also shown to be a crucial regulator of OPC differentiation by means of shifting oligodendroglia toward an astrocytic fate, thereby causing astrogliosis and insufficient remyelination in Theiler’s murine encephalomyelitis [78]. Given that STAT3 pathway can be activated by IFN-γ, the expression of which is increased in DS mice, it needs to be pointed out that IFN-γ was demonstrated to decrease rat OPC differentiation into oligodendrocytes [47]. Moreover, this study also indicated that IFN-γ might shift cell commitment toward the astrocytic lineage. Accordingly, transgenic mice overexpressing IFN-γ under control of the MBP promotor exhibited hypomyelination accompanied by an increase of astrocyte numbers, as well as reactive gliosis in white matter tracts [79]—a shift in brain cytoarchitecture that is strikingly similar to DS neuropathology. Therefore, overstimulation of JAK-STAT signaling caused by overexpressed levels of INFRs, ligands, and subsequent overactivation of the STAT3 pathway may promote an NSC/OPC fate toward an astrogliogenic pathway in the DS brain.

### 4.2. SHH Signaling

The spatiotemporal activity of Sonic Hedgehog (SHH) controls cell proliferation, migration, fate and differentiation of progenitor cell waves during brain development [80]. SHH is a well-known regulator that promotes oligodendroglial fate, OPC generation, differentiation, and myelin production in the spinal cord and forebrain during embryonic development [81,82], as well as OPC production and recruitment throughout adulthood [83] and in demyelination [84]. In the canonical SHH pathway, in the absence of SHH, the inhibitory transmembrane receptor Patched1 (Ptch1) suppresses the activity of the SHH signaling activator Smoothened (Smo) [85]. SHH binding to Ptch1 interrupts its inhibition on Smo, which triggers a complex intracellular signaling cascade including the transcription factors of the Glioma-associated oncogene (Gli) family to mediate downstream gene transcription, such as Mammalian achaetescute homolog-1 (Ascl1/Mash1), Olig2, or Nk2 homeobox 2 (Nkx2.2) [86,87,88]. Ptch1 was shown to be overexpressed in 17–21 week old fetuses and the DS mouse Ts65Dn [89], leading to the assumption that the SHH pathway is repressed in DS. Indeed, Gli 1 and 2, as well as Mash1, are downregulated in trisomic neuronal precursor cells (NPCs) of Ts65Dn mice, which could be restored by the maintenance of SHH signaling activity by Smoothened Agonist (SAG) treatment [73].

Of note, inhibition of Gli1 activity was previously shown to be important for NSC-dependent remyelination [90]. Furthermore, OPC differentiation was shown to be defective and diminished in SHH^−^/^−^ mutants [87,91] and rat OPCs treated with the steroidal alkaloid cyclopamine, which inhibits SHH signaling by targeting Smo [92]. Thus, the increased inhibition of Smo due to elevated Ptch1 levels in DS, subsequently leading to repressed SHH activity, may contribute to the observed downregulation of a whole cluster of genes associated with OPC differentiation and myelination [11] and the delayed differentiation of OPCs, subsequently leading to hypomyelination in DS brains [18].

### 4.3. Notch Signaling

Notch signaling was shown to cross-talk with JAK-STAT [72] and SHH signaling pathways [93], thereby inducing gliogenic shift during brain development. Mediated by binding of ligands such as delta-like protein 1 (Dll1), cleavage of the transmembrane receptor Notch by γ-secretase is initiated. This liberates the Notch intracellular domain (NICD), which translocates to the nucleus to transcriptionally activate Notch effector proteins, such as hairy/enhancer of split 1 and 5 (Hes1, Hes5) [94], which are proteins that were shown to promote the activation of STAT3 [95]. Notch1, Notch2, and Dll1 expression were demonstrated to be significantly upregulated in adult DS fibroblasts and cortices [94]. This process may increase Hes protein activity, thus contributing to the activation of STAT3 and enhancing astroglial differentiation. Notably, Wu and colleagues demonstrated, independently of DS studies, that Notch1 overexpression in glial restricted precursor cells (GRPs) upregulated *Hes1* mRNA levels and that overexpression of Hes1 promoted astrocyte generation at the expense of oligodendrocytes [96]. Taken together, the upregulation of JAK-STAT and Notch signaling may synergistically contribute to astrogliogenesis, thereby suppressing neurogenesis [6] and oligodendrogenesis in the DS brain.

### 4.4. Wnt/β-Catenin Signaling

The wingless and integration site (Wnt) signaling pathway is another fundamental mechanism that directs cell proliferation, polarity, and fate determination during embryonic development and tissue homeostasis [97,98]. Activation of the canonical Wnt pathway is dependent on the nuclear translocation of β-catenin, which drives the expression of several target genes [99]. The canonical Wnt signaling consists of extracellular Wnt proteins/ligands, surface membrane frizzled receptors (Fzd), low density lipoprotein (LDL) receptor related protein-5 and 6 (LRP-5/6), cytoplasmic β-catenin, and intranuclear transcription factors of the T cell factor/lymphoid enhancer factor (TCF/LEF) family. Binding of a Wnt ligand to Fzd and Lrp5/6 causes the degradation of the β-catenin destruction complex, which consists of adenomatous polyposis coli (APC), axin, glycogen synthase kinase 3 β (Gsk3β), and casein kinase 1 (CK1). This leads to the accumulation of β-catenin in the cytoplasm, which then translocates to the nucleus where it induces the expression of downstream target genes, including cyclin Dl, which is mediated by binding to TCF4 [99]. Signaling via the Wnt/β-catenin pathway is also a key regulator of oligodendrocyte development, as it is transiently activated in OPCs concurrent with the initiation of terminal differentiation [100]. β-catenin activity is down-regulated in mature oligodendrocytes, which is necessary for accurate oligodendrocyte differentiation [100], as mutant mice with elevated Wnt/β-catenin signaling in the oligodendrocyte lineage display blocked differentiation and hypomyelination [101]. Paradoxically, however, deletion of the Wnt effector TCF4 does not cause precocious oligodendrocyte differentiation as may be expected, but rather blocks oligodendrocyte differentiation [100,102,103]. Interestingly, loss of β-catenin in NPCs was demonstrated to cause precocious specification and differentiation to astrocytes [104].

In the context of DS, general downregulation of the Wnt/β-catenin signaling pathway was demonstrated in human DS and the DS mouse Tc1 hippocampus [99]. In particular, free, and thus activated, β-catenin levels were dramatically diminished. Contrary to this finding, Li and colleagues observed elevated β-catenin signaling in gene perturbation studies targeting a specific Hsa21-endcoded gene that they suggested was implicated in DS pathogenesis, which nevertheless resulted in defective neurogenesis [105]. Taken together, the aberrant Wnt/β-catenin signaling observed in DS may also contribute to defective oligodendrogenesis and lead to a gliogenic cell-fate shift during early brain development and homeostasis.

### 4.5. Nfatc/Calcineurin Signaling

The nuclear factor of activated T cell (Nfat) pathway is an essential regulator of vertebrate development, which is necessary for the regulation of proliferation and differentiation of NPCs from the SVZ [106]. Activated by calcineurin, a calcium and calmodulin-dependent serine/threonine protein phosphatase, cytoplasmic Nfatc is dephosphorylated and subsequently translocated into the nucleus, where it regulates protein expression such as IL-2 [6]. Regulator of calcineurin 1 (RCAN1), formerly known as DS critical region 1 (DSCR1), inhibits the calcineurin-mediated activation of Nfatc. Interestingly, RCAN1 is located on Hsa21 and was shown to be overexpressed in the DS fetal brain and in Ts1Cje and Ts65Dn mice [107,108]. Accordingly, Nfatc4 was reported to be hyperphosphorylated in the human fetal DS brain at gestation week 20 [109]. In this context, it needs to be mentioned that DYRK1A and RCAN1 were shown to act synergistically to control phosphorylation levels of Nfatc [109,110]. DYRK1A increases RCAN1 inhibitory activity by phosphorylating it and is capable of reducing Nfatc transcriptional activity by directly phosphorylating Nfatc proteins.

Notably, Nfat/calcineurin signaling was shown to be required for oligodendroglial differentiation and myelination by transcription factor network tuning [111]. When Nfatc activation was inhibited by preventing calcineurin binding to Nfats, OPC maturation and differentiation was strongly reduced. This pathway may therefore contribute to aberrant oligodendrogenesis and hypomyelination in DS, as inhibition of Nfatc/calcineurin signaling is mediated by elevated RCAN1 and DYRK1A activities.

### 4.6. APP-Mediated Signaling

The Hsa21-encoded *Amyloid precursor protein* (*APP*) gene is involved in cell migration and cell-cycle progression in brain development [112], specifically influencing NPC proliferation, cell-fate specification, and maturation [113]. Depending on the APP processing pathway (non-/amyloidogenic), APP is cleaved by α-, β-, and γ-secretases, resulting in N-terminal soluble secreted APP (sAPP) and C-terminal fragments, such as Aβ and the APP intracellular domain (AICD). The dysregulation of APP due to triplication was suggested to result in early-onset AD-like pathology in DS. Indeed, APP protein levels were shown to be increased in homogenates from the temporal cortex of fetuses with DS [6,114], and neuritic Aβ plaque formation is present in the hippocampus and enthorinal cortex of almost all adults with DS and in some DS children [115,116,117]. Furthermore, the triplication of APP in Ts65Dn mice was demonstrated to impair NPC proliferation, differentiation and maturation due to increased levels of AICD [73,89,118].

Notably, elevated levels of AICD increased the Ptch1 expression in trisomic NPCs [89], hence the APP/AICD system may at least contribute to the derangement of SHH signaling, as outlined above. Moreover, increased AICD levels can promote Gsk3β activity, thereby reducing the translocation of β-catenin to the nucleus, which may contribute to the suppression of the Wnt/β-catenin pathway [118]. Interestingly, a study in the field of intraventricular hemorrhage (IVH), a common neurological complication of prematurity causing cognitive deficits and ID [119], which is accompanied by inhibited proliferation/maturation of OPCs and hypomyelination [120], demonstrated that Gsk3β activity interfered with OPC differentiation and myelination [121]. Furthermore, a cross-talk of Gsk3β and Notch signaling was shown, as inhibition of Gsk3β downregulated Notch signaling. Accordingly, it can be suggested that increased Gsk3β activity due to APP overexpression may contribute to increased Notch signaling, thus enhancing astrogliogenesis at the expense of oligodendrogenesis. Indeed, exposure to soluble APP was demonstrated to regulate human NPC differentiation through activation of JAK-STAT and Notch signaling and to induce astrocytic differentiation [122]. As Aβ itself was reported to increase apoptosis in oligodendroglia in vitro [123], a more widespread implication of this protein is suggested to lead to aberrant oligodendrogenesis in DS.

## 5. Regulators of Glia Cell Fate: Avenues to Adjust Aberrant White Matter?

Adjusting glia cell-fate imbalance, hence overcoming intrinsic defects in oligodendroglial cell maturation and subsequently developmental dysmyelination, will be a major target in order to improve white matter structures in DS. To this end, repurposing pre-existing modulators or compounds developed for the promotion of endogenous oligodendroglial cell maturation in demyelinating diseases such as multiple sclerosis (MS) [9,124,125,126] represents a possible strategy. In this context, currently evaluated drugs related to the development of myelin-repair therapies are discussed here.

Modulating the Wnt/β-catenin pathway by means of indometacin, a non-steroidal anti-inflammatory drug (NSAID) [127], or Gsk3β inhibitors, such as CHIR99021 or LY-294002, exhibited the potential to promote oligodendrogenesis in healthy and demyelinated paradigms [86,87]. Furthermore, the antifungal agent miconazole, which interferes with ergosterol synthesis, as well as corticosteroid betamethasone clobetasol, which suppresses inflammatory responses, were demonstrated as therapeutic compounds for enhancing (re)myelination in vivo and in human OPCs in vitro [128]. Moreover, modulation of histamine receptor signaling by means of GSK239512, a histamine H3 receptor antagonist, was demonstrated to boost oligodendroglial differentiation as indicated by phenotypic screening and genetic association of human demyelination lesion samples of patients with MS [129]. In this regard, magnetization transfer ratio (MTR)-based post-hoc analyses indicated a small mean improvement in myelin content in treated patients with relapsing remitting (RR) MS relative to placebo [130]. On the other hand, the first-generation histamine H1 receptor blocker clemastine was initially identified as a remyelinating drug in a high-throughput screening [131] and was further investigated in a RRMS clinical study demonstrating a reduction in P100 latency delay in visual evoked potentials (VEPs) [132]. This readout could be used to monitor myelination dependent signal propagation in the visual system. In addition to these OPC-directed drugs, several experimental compounds have been described to trigger signaling pathways modulating oligodendrogenesis. The endothelin (ET) receptor antagonist BQ788 was demonstrated to block endothelin-B receptor activation on astrocytes, thereby rescuing oligodendrogenesis and promoting remyelination [133]. The flavonoid molecule quercetin leads to enhanced oligodendrogenesis and remyelination in several ways, as it suppresses Notch signaling by inhibiting γ-secretase activity and disrupts the binding of β-catenin to TCF4 [134]. In a recent study by Granno and colleagues, a major role of Wnt/β-catenin signaling in DS was implicated. They combined bioinformatics with RNA and protein analyses using post-mortem tissue from adult DS individuals. Among other molecules, they identified axin2 to be significantly decreased in DS [99]. As the small molecule XAV939 was previously shown to stabilize axin2 by inhibiting the poly-ADP-ribosylating enzymes tankyrase 1 and 2 in hypoxic and demyelinating injuries, thereby accelerating OPC differentiation and myelination [135], it might also constitute a possible treatment approach for white matter deficits in DS.

Moreover, small molecule approaches addressing transcriptional/epigenetic regulators affecting oligodendrogenesis could also provide additional therapeutic perspectives. In this regard, GANT61, a blocker of the transcription factor Gli1, was demonstrated to promote the generation of oligodendrocytes from adult NSCs [90]. In accordance, specific inactivation of SIRT1 by means of the small molecule inhibitor EX-527, a protein deacetylase implicated in energy metabolism, increased the production of new NSC-derived OPCs in the adult mouse brain [136]. Likewise, a similar promoting effect could also be attributed to this molecule in the OPC context [137]. Furthermore, activation of the fibroblast growth factor receptor-3 (FGFR3) signaling was recently shown to redirect the differentiation of SVZ-derived NSCs from neuronal to oligodendroglial lineage, hence, promoting remyelination [138]. In this context, the membrane-bound and the cleaved ectodomains of the klotho protein were observed to be associated with FGFR3 signaling. This protein acts as a co-receptor and was found to modulate the Wnt and IGF pathways, thereby enhancing remyelination in demyelinating animal models [139,140].

However, promoting oligodendroglial maturation and axonal ensheathment might not be sufficient for successful white matter restoration or rescue. Reprogramming or reconverting astroglial to oligodendroglial cells, as well as a preservation of the oligodendroglial lineage by means of genetic or pharmacological approaches, are most likely mandatory for white matter stabilization in DS [9,141]. In 2014, information on FDA-approved drugs/small molecules, suitable for rescuing cognitive impairment due to neurodevelopmental alterations, neurotransmitter imbalances, and neurodegeneration in the Ts65Dn DS mouse, was compiled [5]. Of note, the preclinical evaluations that emerged from this study need to be considered critically, as this mouse model did not reflect all trisomic orthologues in individuals with DS, hence an effective translation to human clinical trials is still unclear. However, beside neurogenic effects, some identified drugs are also likely to foster oligodendrogenesis, thus representing potential therapeutics for enhanced myelin development and stabilization. Among these listed drugs, the selective generic estrogen receptor (ER) β agonist diarylpropionitrile (DPN) could be of interest based on the observation that it confers functional neuroprotection in a chronic experimental autoimmune encephalomyelitis (EAE) mouse model of MS by stimulating endogenous remyelination [142]. The stimulation of glial progenitor cells (GPCs) derived from both the SVZ and white matter with memantine, a low-affinity antagonist of NMDA receptors used to treat AD, was found to promote oligodendrogenesis, and therefore myelin repair, upon ischemic periventricular leukomalacia (PVL) [143]. Moreover, fluoxetine, an antidepressant based on selective serotonin reuptake inhibition, also known as Prozac, was demonstrated to boost oligodendrocyte-related gene transcripts such as *CNPase*, *OLIG1*, and *MOG* when applied to rhesus monkeys with major depressive disorders [144]. Lithium chloride (LiCl), which was established for the treatment of bipolar disorder (BD), can stimulate oligodendrocyte morphological maturation and promote remyelination after toxin-induced demyelination of organotypic slice cultures [145]. In addition, melatonin, a sleep/wake-cycle regulating hormone, was shown to increase oligodendrocyte generation from NSCs [146]. Further to this, the vitamin E derivate TFA-12 was found to reduce astrogliosis and to accelerate remyelination of toxin-induced demyelinated lesions [147]. Given the impact of SHH signaling in oligodendrogenesis, a recent study demonstrated that the small molecule Smo agonist SAG could alter SHH signaling in DS [148]. Notably, SAG modulates oligodendroglial differentiation and additionally steers commitment of NSCs to the oligodendroglial lineage [92]. Interestingly, the γ-secretase inhibitor DAPT (N-[N-(3,5-difluorophenacetyl)-1-alanyl]-S-phenyl-glycinet-butylester) is an effective inhibitor of the Notch signaling pathway and might also confer benefits to white matter, as it was found to promote differentiation of NSCs/NPCs into oligodendrocytes, astrocytes, and neurons in vitro [149]. Nevertheless, to what degree the balance between these two glial cell types is affected remains to be shown. In this context, such a preferred shift toward oligodendroglia could be mediated via the acetylcholine esterase inhibitor donepezil, an FDA-approved drug for Alzheimer’s disease and dementia. Donepezil was shown to promote the differentiation of primary NSCs into mature oligodendrocytes at the expense of astrocytes [150], and it was also found to enhance myelin sheath generation in neuron/glia co-cultures [151]. The FDA-approved anti-seizure drug ethosuximide was described to be capable of inducing trans-differentiation of muscle-derived stem cells into Olig2-positive oligodendroglial cells [152]. Whether ethosuximide activity could also be used to re-establish the glial cell balance needs to be shown, keeping in mind that Olig2 expression itself is not restricted to oligodendroglial cells, but is also found in the cytoplasm of astroglia [153].

The histone deacetylase class I and II inhibitors trichostatin A and valproate (VPA) were previously demonstrated to promote the conversion of astrocytes to OPCs [154,155], and could thus be considered as potential regulators for the desired glial shift in DS. A similar mode of action was revealed in response to forced expression of the microRNA miR-302/367 cluster, thereby enhancing the generation of oligodendroglia from astrocytes [156]. Likewise, injection of Sox2 lentiviral particles into the corpus callosum following cuprizone-mediated demyelination in vivo as well as lentiviral transduction of astroglial cells in vitro resulted in a conversion of astrocytes to oligodendroglial cells [156]. Similarly, overexpression of pro-oligodendroglial transcription factors, such as Olig2 or Ascl1/Mash1, also resulted in reprogramming of NSCs toward an oligodendroglial fate [157,158,159,160]. Of note, *Olig2* is an Hsa21-encoded gene, which was shown to be overexpressed in DS and assumed to interfere with neurogenesis in DS [6]. However, we demonstrated here that oligodendrogenesis is negatively affected in DS.

Nevertheless, it appears that the drugs and transcriptional/epigenetic regulators described here could indeed provide new avenues for the experimental and clinical rescue of white matter deficits in DS. To what degree some of these candidates are applicable in the context of DS in terms of application and opportunity windows certainly needs additional experimental and pre-clinical research efforts.

## 6. Concluding Remarks

Given the importance of myelinating glial cells for axonal support, trophism, maintenance, and electrical insulation, an overall increase in the number of functional oligodendrocytes would likely confer an overall benefit on neuronal cell numbers and functionality, which in turn could ameliorate ID, even in light of known neurogenic deficits in DS. Strikingly, DS research has so far mainly focused on aberrant neurogenesis and the underlying signaling pathways leading to defective neuronal cell proliferation, differentiation, and progenitor cell fate in DS. In this review, the collected evidence suggests that many of these dysregulated signaling pathways may also be involved in defective DS-related NSC/OPC proliferation, differentiation, and fate commitment. Furthermore, we demonstrated that several drugs and molecules identified to restore brain developmental deficits in rodent DS models based on neurogenesis criteria [5] might also mediate beneficial effects on the oligodendroglial lineage.

Hsa21 is the smallest human chromosome, currently known to encode more than 400 genes. This number might increase over time due to the recognition of non-coding RNAs [161,162]. Nevertheless, the description of dosage-sensitive Hsa21 genes resulting in a specific phenotype by gene-copy number variations is currently limited to only a few. This may be due to the fact that over 20 proteins encoded by Hsa21 are involved in signal transduction and more than 30 proteins are considered to belong to transcription factors, both of which most likely influence the expression of other genes in the genomes of DS patients [7]. By implication, this inevitably results in a genome-wide dysregulation of several networks at the same time, which could be demonstrated, for example, in the case of the gene cluster M43, which is related to oligodendroglial differentiation and myelination [11].

Interestingly, pharmacological approaches addressing neurogenic deficits were found to be successful in a broad age range (prenatal, perinatal, and adult) of treated Ts65Dn mice, suggesting that the prevention or amelioration of cognitive deficits in DS may indeed be possible. This paves the way toward clinical trials, some of which (donepezil, folate or memantine) are still in progress, but no differences in outcome between treated and placebo have occurred as yet [5]. In humans, however, prenatal treatments will be challenging due to specific safety requirements. Nevertheless, the window of opportunity to improve differentiation and homeostasis in the oligodendroglial lineage might stretch over several developmental phases, as myelination is mainly a postnatal event. In this regard, it is worth mentioning that dysregulation of the M43 gene cluster, which is related to oligodendroglial lineage, appears during late neonate’s development and during the first years of postnatal life in DS [11], a period that coincides with massive upregulation of oligodendroglial and myelination genes [163], as well as oligodendrocyte expansion in the human brain [164].

A number of mouse models with DS-related features were generated and used to study Hsa21dosage-sensitive genes and to understand their roles leading to cognitive impairment (reviewed in [5,165,166]). Although well-established mouse models such as Ts65Dn, Ts1Cje, and Ts16 recapitulate the human neuropathological phenotype to a certain extent, modeling of DS in rodent system remains challenging because Hsa21 genes are distributed throughout mouse chromosomes 16, 17, and 10 (Mmu16/17/10). Therefore, mouse models may provide different outcomes, hence negatively affecting translation to humans.
